# Endoscopic ultrasound—assisted rendezvous technique followed by dual-duct drainage using a double-lumen papillotome: a salvage strategy for postendoscopic papillectomy stricture

**DOI:** 10.1016/j.vgie.2026.02.013

**Published:** 2026-03-06

**Authors:** Go Endo, Naminatsu Takahara, Hiroto Nishio, Kensaku Noguchi, Tatsuya Sato, Ryunosuke Hakuta, Yousuke Nakai, Mitsuhiro Fujishiro

**Affiliations:** 1 Department of Gastroenterology, Graduate School of Medicine, The University of Tokyo, Tokyo, Japan; 2 Department of Internal Medicine, Institute of Gastroenterology, Tokyo Women's Medical University, Tokyo, Japan

## Abstract

**Background and Aims:**

Papillary stricture is a clinically relevant late adverse event of endoscopic papillectomy (EP) and may result in concurrent cholangitis and pancreatitis. Although endoscopic drainage is the first-line modality, biliary and pancreatic duct access can be challenging because of fibrotic strictures after EP. This article aims to demonstrate the feasibility of EUS-assisted rendezvous (EUS-RV) combined with a double-lumen papillotome (MagicTome; PIOLAX Inc, Yokohama, Japan) to achieve dual-duct access.

**Methods:**

We present a case of a 70-year-old woman with cholangitis and pancreatitis caused by post-EP stricture. After a failed ERCP, a salvage EUS-RV was performed to access the biliary duct, followed by pancreatic duct cannulation using a double-lumen papillotome.

**Results:**

The distal bile duct was punctured from the duodenum with a 19-gauge needle (EZ Shot 3 Plus; Olympus, Tokyo, Japan), and a 0.025-inch guidewire (VisiGlide 2; Olympus) was advanced through the stricture into the duodenum. The guidewire was retrieved with biopsy forceps, and biliary access was established. Subsequently, using a double-lumen papillotome, we cannulated the pancreatic duct. Balloon dilation and plastic stent placement were performed in both ducts without adverse events. The stents were removed after 12 months. No recurrence was observed during 12 months of follow-up.

**Conclusions:**

EUS-RV combined with a double-lumen papillotome is a useful salvage strategy for complete post-EP strictures. This method can be applied to difficult cannulation, particularly when dual-duct access is required.

## Introduction

Papillary stricture is a clinically relevant late adverse event of endoscopic papillectomy (EP), with a reported incidence of 3.0% to 7.3%,^1^, ^2^, ^3^ and may cause concurrent cholangitis and pancreatitis.^4^ Because post-EP strictures often cause dense fibrotic obstruction, standard ERCP access can be challenging, particularly in cases involving the bile and pancreatic ducts. EUS-assisted rendezvous (EUS-RV) is a promising salvage approach for failed ERCP.^5^ We report a case of post-EP dual-duct strictures successfully managed using EUS-RV in combination with a double-lumen papillotome.

## Case presentation

**Figure 1:**
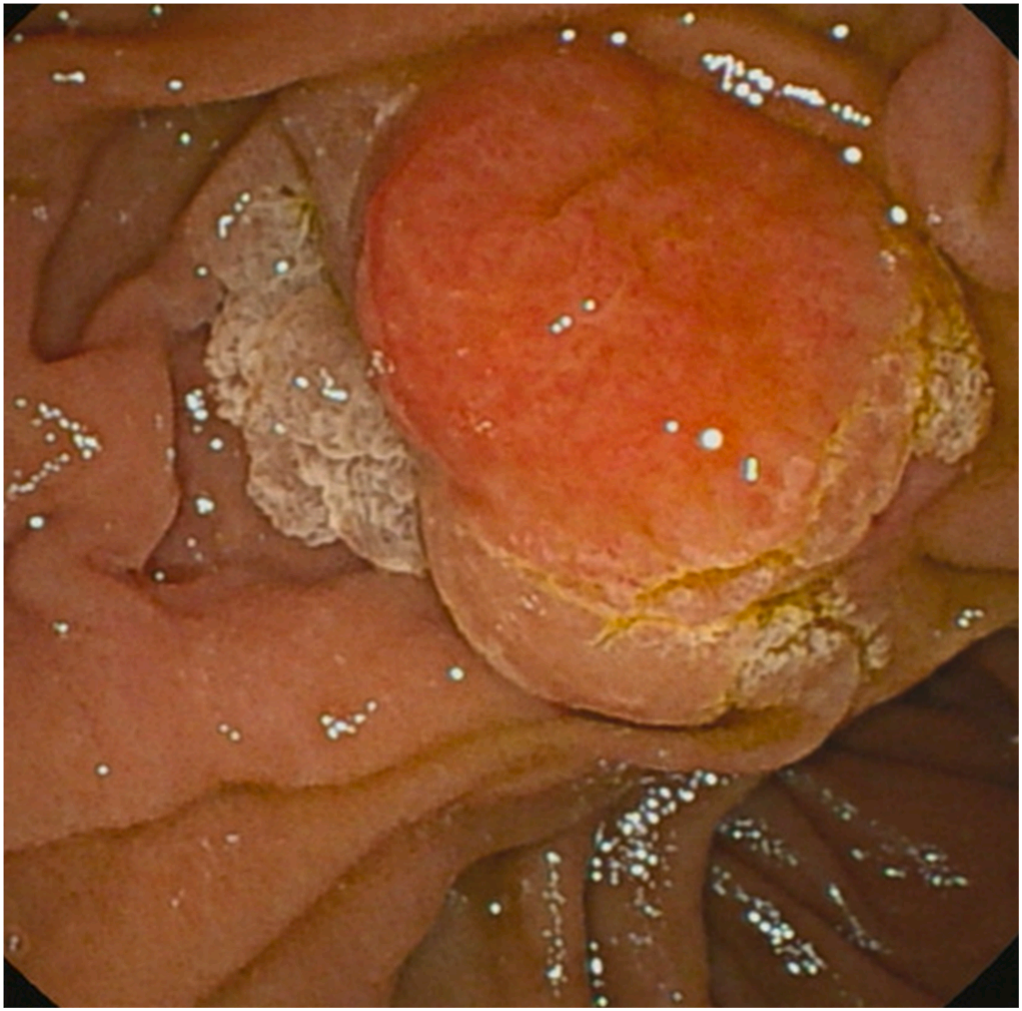
Endoscopic view of the ampullary adenoma. A 25-mm tumor with marked erythema was present at the major papilla, with a laterally spreading lesion of whitish, granular changes in the surrounding mucosa.

**Figure 2:**
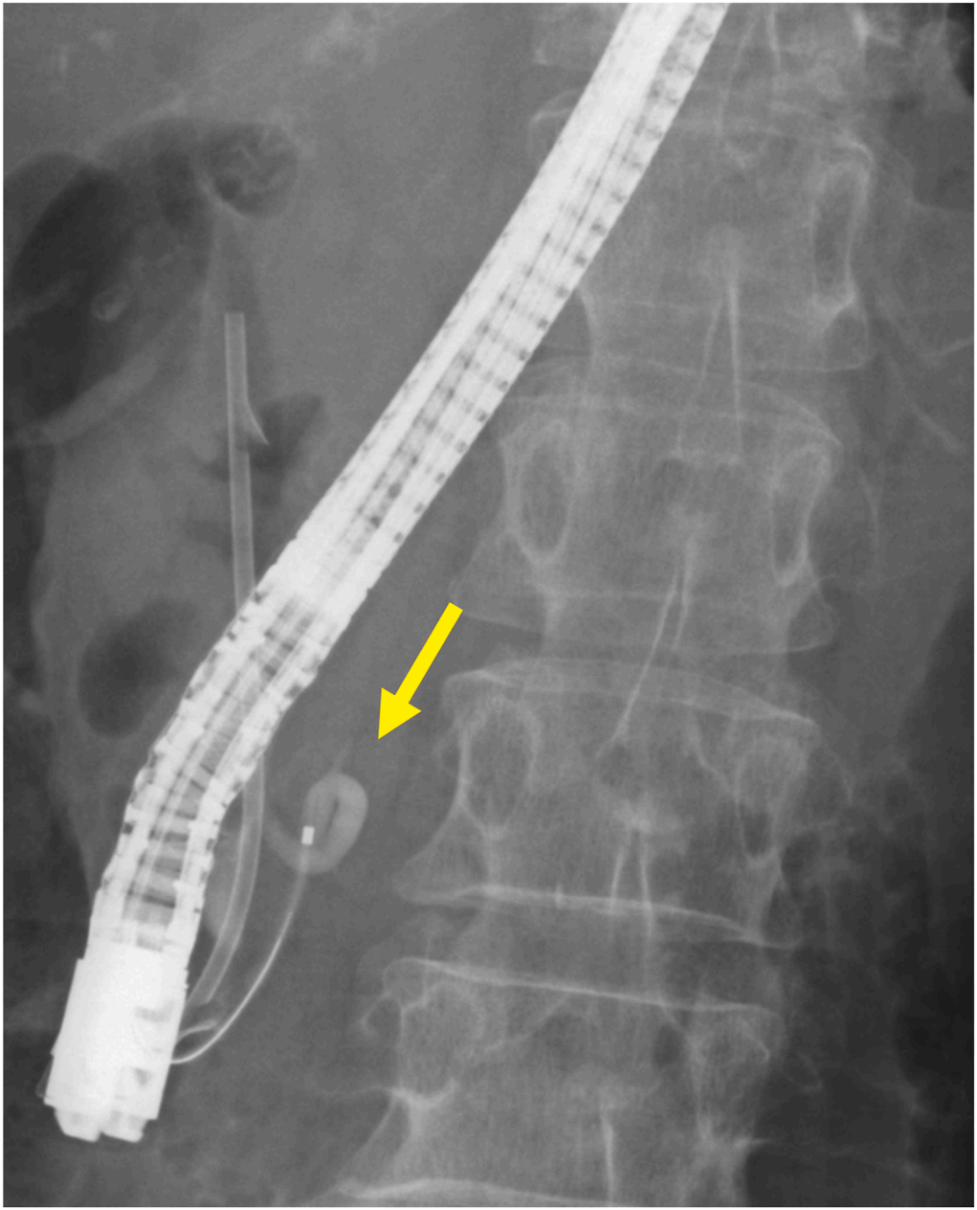
Fluoroscopic image of pancreatogram during endoscopic papillectomy. The main pancreatic duct was looping in the pancreatic head (*arrow*), making pancreatic stent placement difficult.

A 70-year-old woman was referred for endoscopic treatment of a 25-mm ampullary adenoma (Fig. 1). Given the lateral tumor spread, hybrid EP was performed to ensure R0 resection. This technique involved a circumferential incision with partial submucosal injection and dissection, followed by snare resection.^6^ Both lateral and vertical margins were negative for a tumor by en bloc resection. At our institution, prophylactic biliary and pancreatic stents are placed for approximately 1 week to reduce the risk of postprocedural adverse events. In this case, a 7F biliary plastic stent was placed without sphincterotomy; however, pancreatic stent placement was avoided because of a looped pancreatic duct (Fig. 2). The mucosal defect was closed using endoscopic clips, and no early procedure-related adverse events were observed. The patient was discharged without indwelling stents on postoperative day 10.

**Figure 3:**
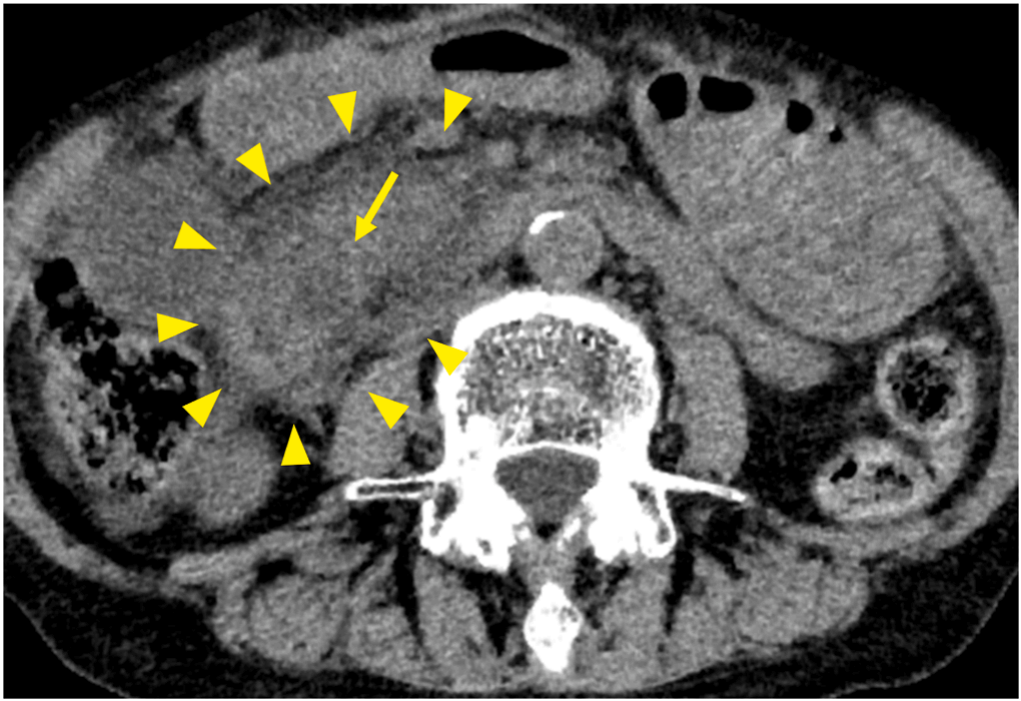
CT image showing swelling of the pancreatic head (*arrowheads*) and dilation of the common bile duct (*arrow*). These findings are suggestive of cholangitis and pancreatitis secondary to postendoscopic papillectomy stricture.

**Figure 4:**
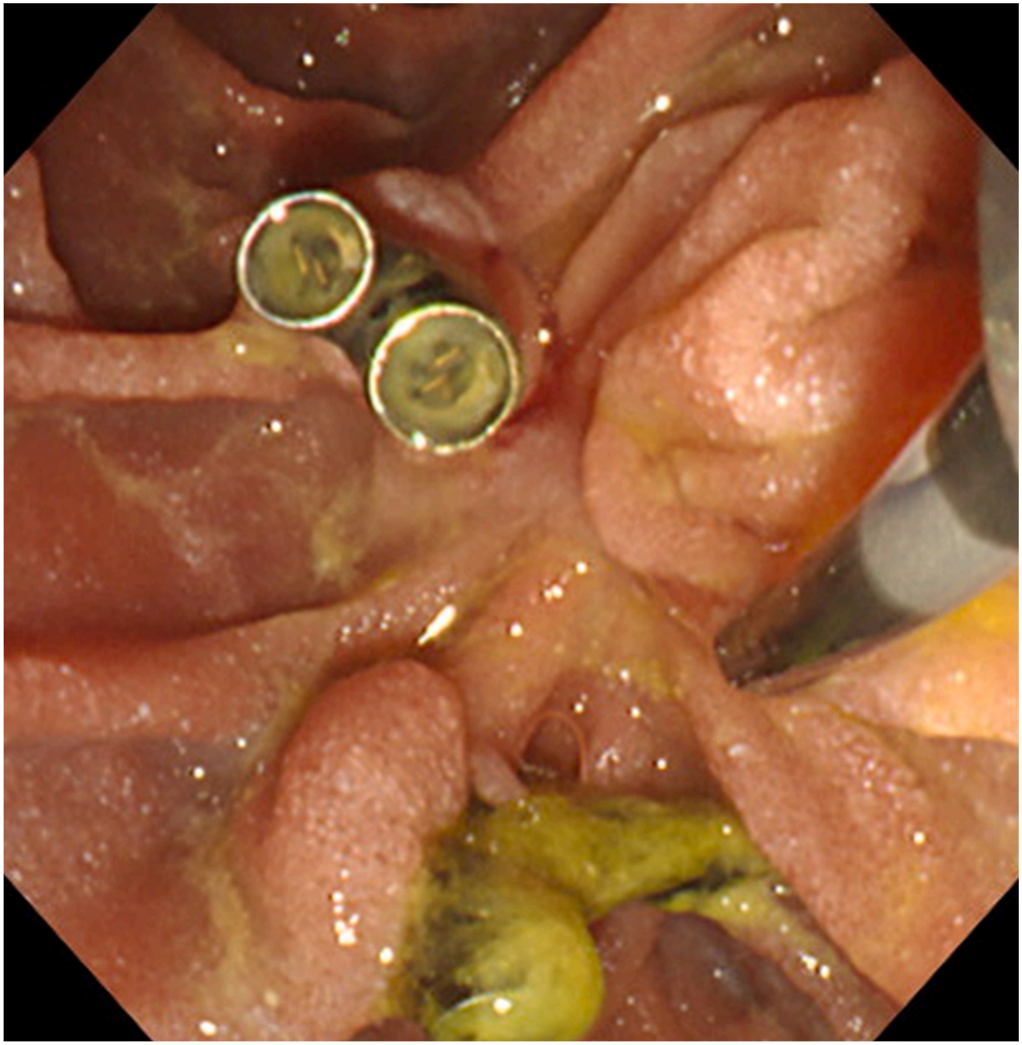
Endoscopic view during ERCP. The papillary orifice is completely strictured by fibrotic tissue.

**Figure 5:**
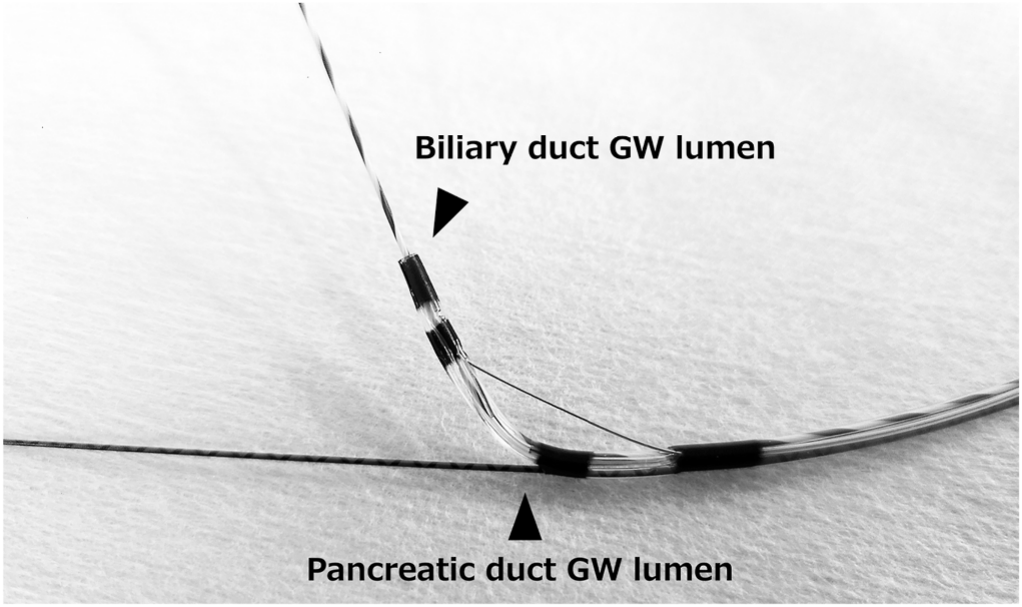
Photograph of the double-lumen papillotome that allows fine adjustment of the insertion angle.

**Figure 6:**
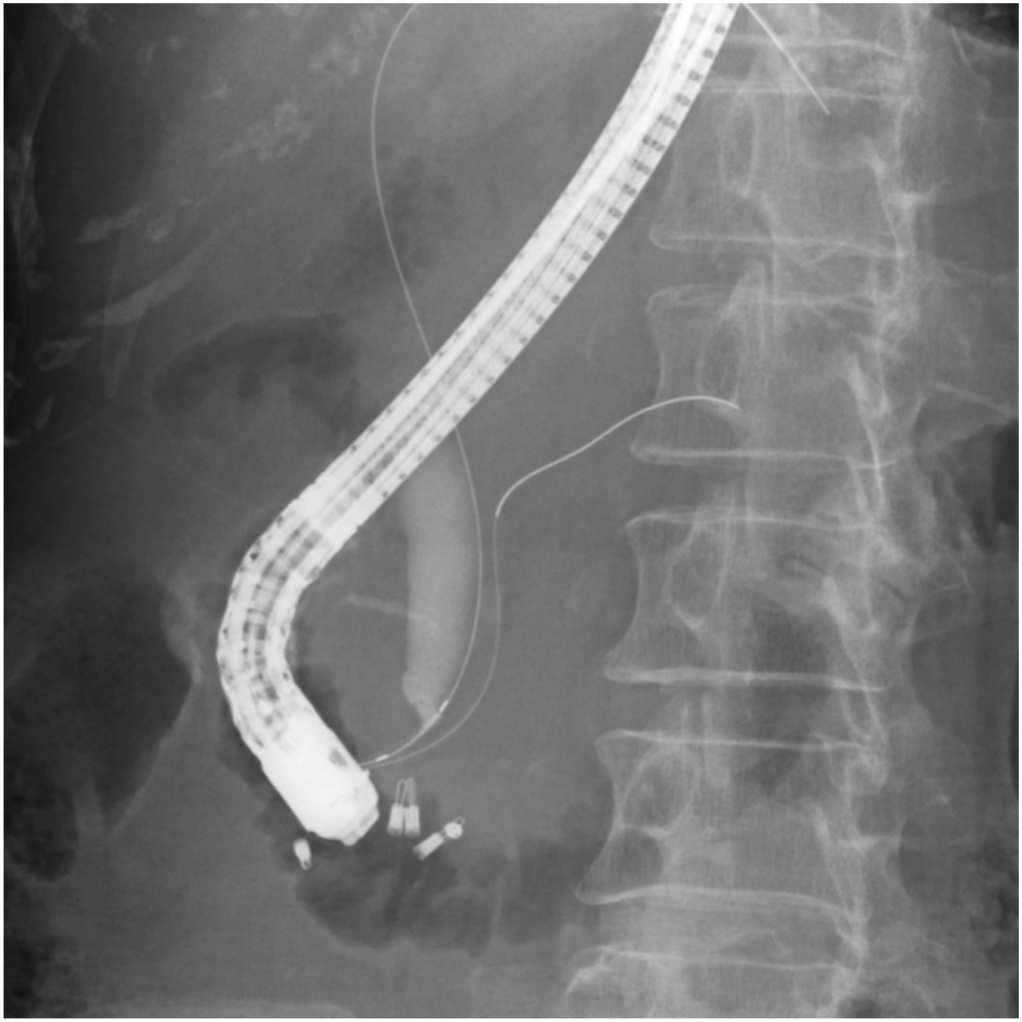
Fluoroscopic view showing guidewires placed in both the bile and pancreatic ducts via the same papillary access after EUS-assisted rendezvous. The previously looped pancreatic duct appears straightened.

**Figure 7:**
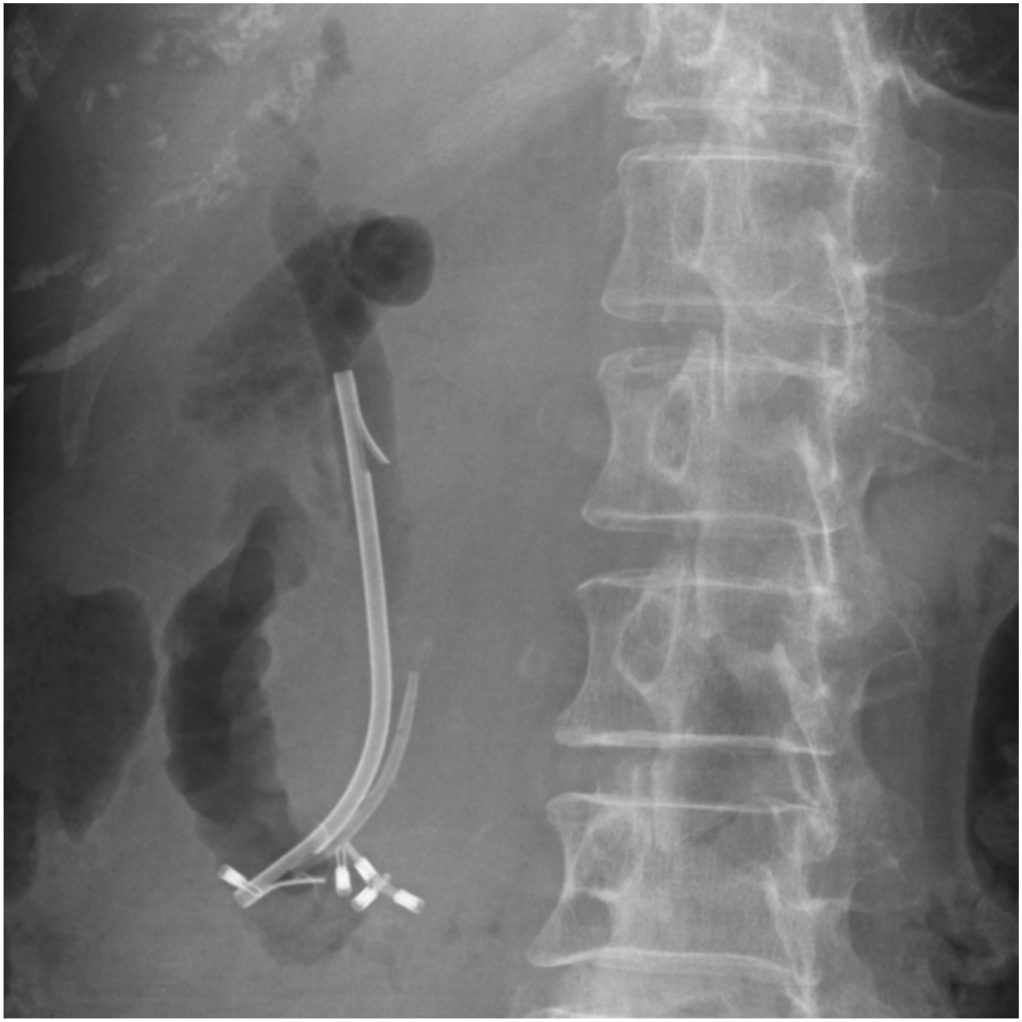
Fluoroscopic image after stent placement. Plastic stents were successfully inserted into both ducts after tract dilation.

Three months later, the patient presented with acute cholangitis and pancreatitis caused by post-EP stricture (Fig. 3). ERCP was unsuccessful because of complete obstruction of the papillary orifice (Fig. 4), and EUS-RV was attempted (Video 1, available online at www.videogie.org). The extrahepatic bile duct was punctured using a linear-array echoendoscope (EG-740UT; Fujifilm, Tokyo, Japan) with a 19-gauge needle (EZ Shot 3 Plus; Olympus, Tokyo, Japan). A 0.025-inch guidewire (VisiGlide 2; Olympus) was initially introduced, but direct advancement into the duodenum failed because of severe stricture. Therefore, the needle was exchanged for an ERCP catheter (MTW; ABIS, Hyogo, Japan), which allowed more precise guidewire manipulation toward the duodenum and successful passage into the duodenum. To prevent guidewire dislodgement during scope exchange, the guidewire was sufficiently coiled within the duodenum. After exchange to a duodenoscope (TJF-Q290V; Olympus), biliary cannulation alongside the guidewire was unsuccessful. Consequently, an over-the-guidewire technique was applied, retrieving the floppy end of the 450-cm guidewire with biopsy forceps while advancing the opposite end to prevent guidewire loss. After biliary cannulation, the floppy end of the guidewire was redirected into the hepatic hilum. Subsequently, pancreatic cannulation was attempted using a double-lumen papillotome (MagicTome; PIOLAX Inc, Yokohama, Japan). This device is equipped with 2 lumens—1 opening at the tapered tip and the other approximately 17 mm proximal to the tip—each accommodating a 0.025-inch guidewire. The device also features a conventional upward-swinging tip for fine control of the insertion angle (Fig. 5). After the papillotome tip was partially inserted into the bile duct and gently bowed upward, the proximal lumen opening was oriented toward the pancreatic duct orifice, and a second guidewire (J-WIRE Prologue; J-MIT, Shiga, Japan) was successfully advanced into the pancreatic duct under assistant manipulation (Fig. 6). Balloon dilation was then performed to 10 mm in the bile duct and 4 mm in the pancreatic duct (REN; Kaneka Medical, Osaka, Japan), followed by plastic stent placement in both ducts (Fig. 7). Guidewire placement straightened the previously looped pancreatic duct, enabling easy stent placement. No procedure-related adverse events occurred, and both cholangitis and pancreatitis resolved promptly.

**Figure 8:**
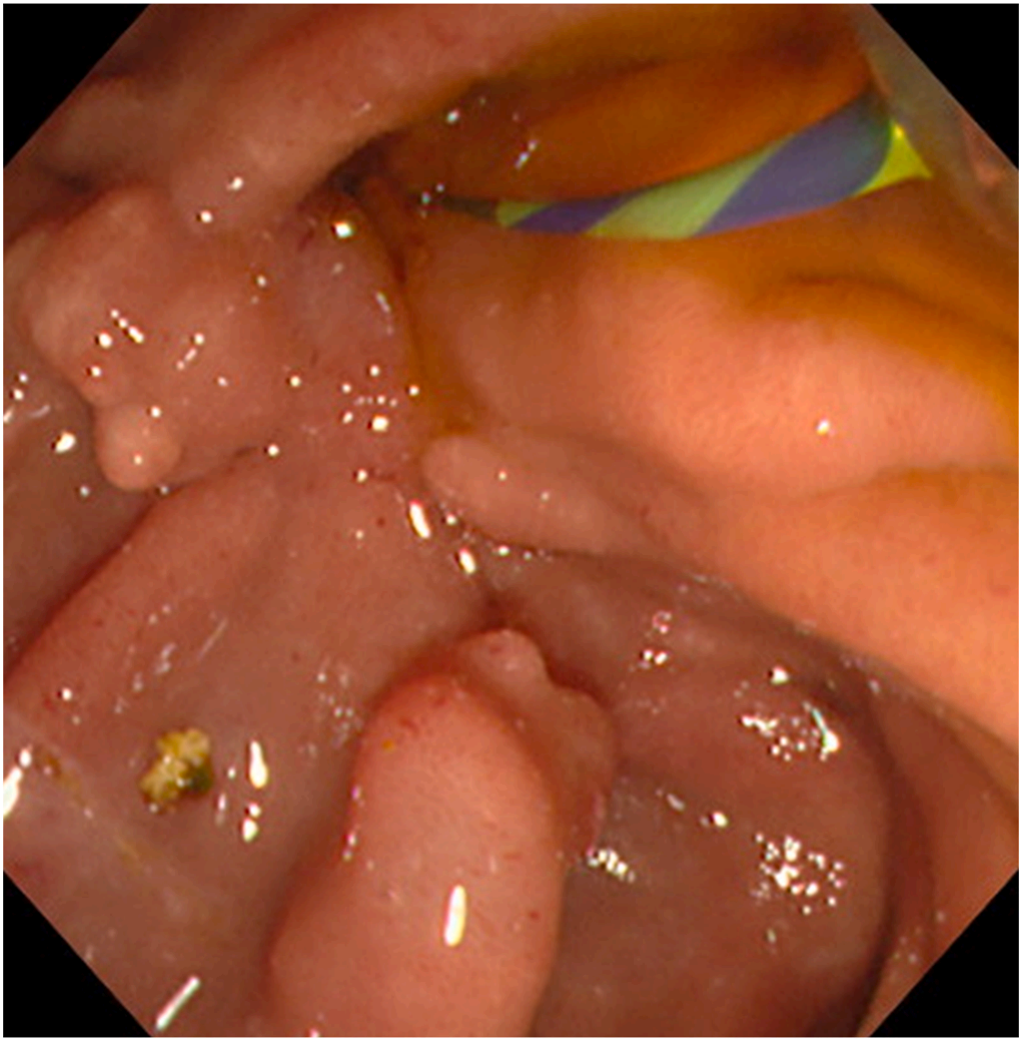
Endoscopic image at 12 months after endoscopic papillectomy. The resolution of the papillary stricture was confirmed at this time, and both stents were removed.

**Figure 9:**
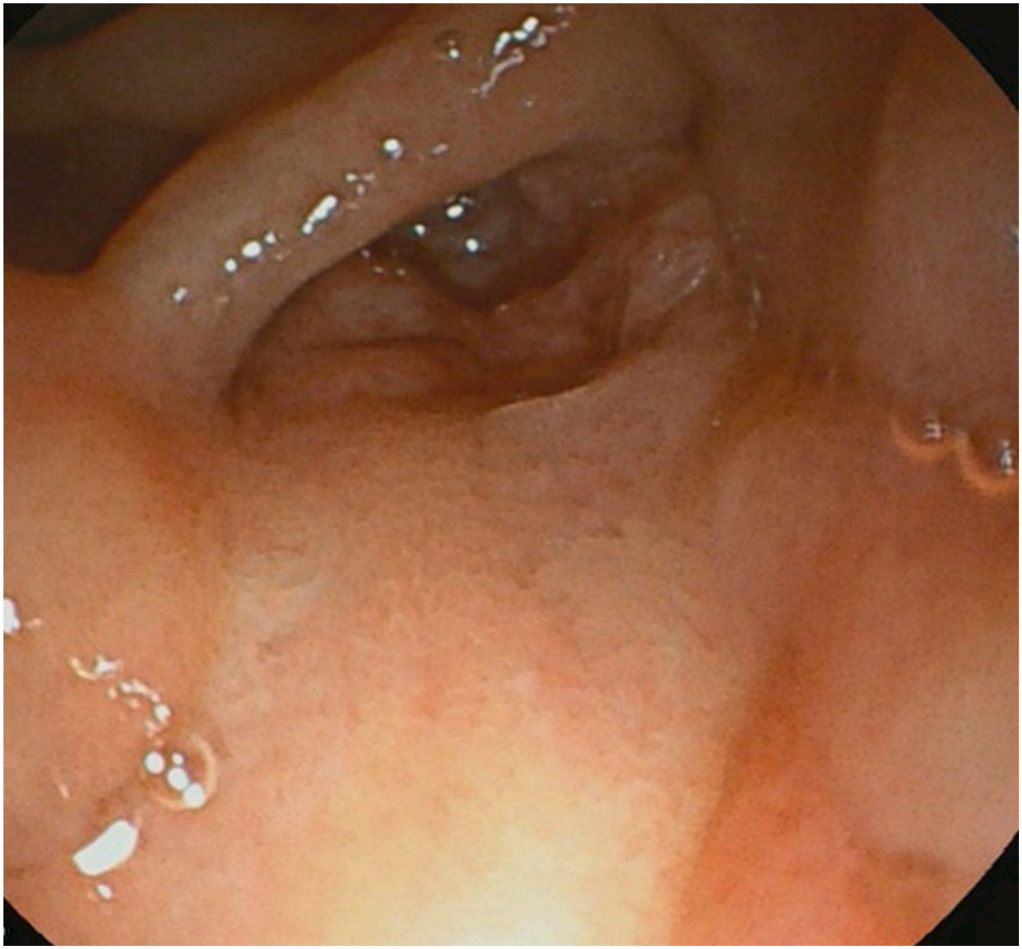
At 12 months after stent removal, endoscopy revealed no evidence of recurrence.

Scheduled stent exchange and/or additional stent insertion with dilation were repeated at 4 and 8 months. A total of three 10F plastic stents were placed in the bile duct, whereas a single 7F stent was placed in the pancreatic duct. At 12 months, all stents were removed after confirming resolution of the stricture (Fig. 8). Endoscopic evaluation at 12 months after stent removal showed no recurrence (Fig. 9), and the patient remained asymptomatic.

## Conclusions

This case highlights the utility of EUS-RV as a salvage approach for failed ERCP with post-EP dual-duct strictures. Although the utility of a double-lumen papillotome for difficult biliary cannulation has been reported,^7^ this case extends its application to achieving dual-duct access a single biliary puncture.

## Patient consent

Informed consent was obtained from the patient for the procedure and publication of this article, including all images and video content.

## Ethical statement

Institutional Review Board approval was waived for this single-case report in accordance with institutional policy.

## Disclosure

The following authors disclosed financial relationships: N. Takahara: Research grant from 10.13039/501100002424Fujifilm. Y. Nakai: Research grant from 10.13039/501100002424Fujifilm; honoraria from 10.13039/100016242Boston Scientific Japan. M. Fujishiro: Honoraria from Olympus Marketing. All other authors disclosed no financial relationships.

## Supplementary data

Video 1 EUS-assisted rendezvous procedure after post-endoscopic papillectomy stricture. A transbiliary approach allowed dual access to the bile and pancreatic ducts via a single puncture site.
